# Dysfunctional lactate metabolism in human alveolar type II cells from idiopathic pulmonary fibrosis lung explant tissue

**DOI:** 10.1186/s12931-021-01866-x

**Published:** 2021-10-28

**Authors:** Danforth A. Newton, Robyn G. Lottes, Rita M. Ryan, Demetri D. Spyropoulos, John E. Baatz

**Affiliations:** 1 Department of Pediatrics, Medical University of South Carolina, Charleston, SC 29425 USA; 2 Department of Pediatrics, Case Western Reserve University, UH Rainbow Babies and Children’s Hospital, Cleveland, OH 44106 USA; 3 Department of Pathology and Laboratory Medicine, Medical University of South Carolina, Charleston, SC 29425 USA; 4 Department of Pediatrics/Division of Neonatology, Medical University of South Carolina, 165 Ashley Avenue, MSC 917, Charleston, SC 29425 USA

**Keywords:** Idiopathic pulmonary fibrosis, Alveolar type II epithelial cells, Lactate metabolism, Mitochondria, Lactate dehydrogenase

## Abstract

**Background:**

Idiopathic Pulmonary Fibrosis (IPF) is the most common and progressive form of the interstitial lung diseases, leading most patients to require lung transplants to survive. Despite the relatively well-defined role of the fibroblast in the progression of IPF, it is the alveolar type II epithelial cell (AEC2) that is now considered the initiation site of damage, driver of disease, and the most efficacious therapeutic target for long-term resolution. Based on our previous studies, we hypothesize that altered lactate metabolism in AEC2 plays a pivotal role in IPF development and progression, affecting key cellular and molecular interactions within the pulmonary microenvironment.

**Methods:**

AEC2s isolated from human patient specimens of non-fibrotic and IPF lungs were used for metabolic measurements, lactate dehydrogenase (LDH) analyses and siRNA-mediated knockdown experiments.

**Results:**

AEC2s isolated from human IPF lung explant tissues had lower rates of oxidative metabolism and were more glycolytic lactate-producing cells than were AEC2 from control, non-fibrotic lung explant tissues. Consistent with this shift in metabolism, patient-derived IPF AEC2s exhibited LDH tetramers that have higher ratios of LDHA:LDHB (i.e., favoring pyruvate to lactate conversion) than control AEC2s. Experimental manipulation of LDHA subunit expression in IPF AEC2s restored the bioenergetic profile characteristic of AEC2 from non-fibrotic lungs.

**Conclusions:**

These results are consistent with the concept that altered lactate metabolism may be an underlying feature of AEC2 dysfunction in IPF and may be a novel and important target for therapeutic treatment.

## Introduction

Idiopathic pulmonary fibrosis (IPF) is the most common form of interstitial lung disease, characterized by progressive and extensive formation of fibrous tissue in the pulmonary parenchyma. Very few treatment options and no therapeutic agents are available that have significant impacts on disease course, ultimately leading most patients to need lung transplants. Annual medical costs for IPF in the U.S are estimated to be $2 billion [1]. The average age at IPF diagnosis is 65 years, consistent with studies suggesting that environmental chemical exposure plays a significant role in disease initiation and/or progression; however, viral infections, gastroesophageal reflux disease, or genetic predisposition could play roles as well [2–5].

Fibroblasts have been the focus of IPF progression studies [6, 7]; however, alveolar type II epithelial cells (AEC2) are gaining recognition as playing important roles in exacerbating the damage that promotes the disease [8, 9]. In this paradigm, injured and apoptotic AEC2s produce pro-fibrotic signals that stimulate fibroblast activation and conversion to the myofibroblast phenotype [9, 10]. In turn, myofibroblasts produce excessive amounts of extracellular matrix molecules, disrupting epithelial-fibroblast cell–cell signaling that further damages the alveolar epithelium and promotes epithelial-to-mesenchymal transition wherein the AEC2 also acquire a fibroblast phenotype [11–13]. However, the initial causes of AEC damage remain poorly understood, and the underlying mechanisms of IPF progression remain insufficiently addressed.

Notably, lactic acid is significantly elevated in IPF vs. healthy lung tissue by approximately threefold [14], a finding consistent with altered cellular metabolism and/or clearance of lactate. The authors of that work proposed the mechanism that increased lactate production, likely by fibroblasts, may lead to pH-dependent activation and release of TGFβ, further promoting the myofibroblast phenotype in IPF. Numerous gene-related changes found in IPF, including mTOR activation and miR200 family downregulation, are known to increase expression of the lactate dehydrogenase (LDH)-A subunit which drives lactic acid production, and LDHA activity has been proposed as a target for IPF therapy [13, 15–17]. Taken together, these studies led us to directly examine AEC2 from IPF patient lungs to determine if these cells also contribute to the increased production of lactic acid associated with IPF.

We have previously shown that healthy AEC2s are highly oxidative and preferentially utilize lactate as a metabolic substrate for mitochondrial ATP production [18]. As presented in this report, we examined and compared metabolic profiles of AEC2s isolated from IPF and non-fibrotic patient explant lung tissues to test the hypothesis that lactate metabolism is altered in IPF AEC2s. Measurements of oxidative phosphorylation and glycolysis were performed, as were profiles of LDH isozymes, the latter of which is an indicator of lactate utilization and production [19, 20]. We also experimentally manipulated cellular LDH isozyme composition which, combined with other results, strongly suggests that the bioenergetic profiles of AEC2s from IPF patients are dysregulated compared to those of non-fibrotic lungs and are greatly dictated by altered LDH isozyme profiles. These studies propose that altered LDH expression in AEC2s may be a novel target for IPF therapies.

## Methods

### Human AEC2 primary cell isolation and culture

Lung samples were obtained with approval of the Medical University of South Carolina (MUSC) Institutional Review Board for Human Research. Non-fibrotic, control lung samples consisted of excess tissue deemed normal by a pathologist and obtained from lobectomies in collaboration with the Hollings Cancer Center Biorepository at MUSC. IPF samples were obtained from surgical lung transplant explants. Specimens were preserved as < 1 cm^3^ pieces by cryoprotectant infusion (CRX-SK; Cryogenix, LLC, Charleston, SC) and storage until experimental use as previously described [21]. Modifications of a published protocol [22] were used to purify primary AEC2s (CD31^neg^, CD14^neg^, CD45^neg^, Ep-CAM^pos^) from lung explant tissue by magnetic bead sorting. Briefly, five to ten < 1 cm^3^ cryopreserved lung pieces were thawed and minced, then dissociated in dispase plus 300 U/mL collagenase Ia (Stem Cell Technologies, Cambridge, MA, and Sigma-Aldrich, St. Louis, MO, respectfully). AEC2s were then purified by magnetic sorting using antibody-coated MicroBeads and MACS Cell Separation system (Miltenyi Biotec, Auburn, CA). Negative selection was performed using a mixture of MicroBeads (coated with anti-CD45, -CD14, and -CD31 antibodies), followed by positive selection on a second magnetic column using anti-CD326 Microbeads (Ep-CAM; all Microbeads from Miltenyi Biotec). Cell viability was assessed using trypan blue dye exclusion. An aliquot of cells was checked for AEC2 purity by modified PAP staining of lamellar bodies [23]. For experimental use, a cell purity threshold of > 85% AEC2 was used. For immunocytochemistry, AEC2 cells were plated on slides, fixed with 4% paraformaldehyde, permeabilized with 0.25% Triton X-100, labelled with 1:2000 anti-pro-SPC (Millipore AB3786) in 10% donkey serum; followed by Alexa Fluor 488-conjugated, donkey anti-rabbit secondary antibody (Invitrogen). TO-PRO-3 (Invitrogen) was used for nuclear staining, and an isotype control primary antibody (ThermoFisher) was used to establish background staining. Images were obtained using a Zeiss Axio Imager 2 (Carl Zeiss Microscopy, White Plains, NY).

Cells were seeded in tissue-culture plates pre-coated with a thin gel of 30% rat-tail collagen (Sigma-Aldrich)/70% EHS (Matrigel, BD Biosciences, San Jose, CA)and incubated in DMEM/F-12 medium (Life Technologies, Grand Island, NY) containing 5.5 mM d-glucose, 1 mM sodium lactate, SAGM supplement (Lonza, Walkersville, MD), and 10 ng/mL keratinocyte growth factor (R&D Systems, Minneapolis, MN). For some experiments, the human adenocarcinoma cell-line A549 [24] was also used and cultured in the same conditions.

### Measurement of AEC2 cellular O_2_ consumption rates (OCR) and proton production rates (PPR)

OCR and PPR in AEC2 primary cells were measured by extracellular flux analyses using a Seahorse Bioscience XF96 instrument (North Billerica, MA), as previously described [25]. Metabolic measurements were compared between primary AEC2s isolated from 3 non-fibrotic human lung tissues (con), AEC2s from explant lung tissues from 3 IPF transplant patients, and the A549 cell-line. Each AEC2 sample (or A549 cells) were analyzed in quadruplicate replicates. The day before measurements, AEC2s (or A549 cells) were seeded in 96-well XF96 assay plates pre-coated with rat-tail collagen at 2.5–5.0 × 10^4^ cells/well in culture medium described above. Before extracellular flux analyses, medium was removed and replaced with unbuffered XF Base Medium (Seahorse Bioscience) formulated with 5.5 mM glucose, 1 mM lactate, and 10 µg/mL insulin. Data were transformed using “Level (Direct) AKOS” algorithm [26] using the Seahorse XF96 software package. Upon assay completion, cells were lysed, and values were normalized to well protein content as determined by bicinchoninic acid assay (Sigma-Aldrich).

### LDH isoenzyme assay

Comparative expression of LDH tetramer isoforms in cellular lysates was determined by native gel electrophoresis and nitroblue tetrazolium staining [27, 28]. Cells were lysed on ice in non-denaturing Native Lysis Buffer (Abcam, Cambridge, MA) containing 1X protease-inhibitor cocktail (Sigma-Aldrich) before separation on 1.5% agarose gels (Agarose-1000, Life Technologies) formulated in 25 mM Tris/250 mM glycine (pH 9.5). Five purified LDH isozymes (LDH-1, -2, -3, -4 and -5; Aalto Scientific, Eatonton, GA) were run separately on each gel to verify identification of LDH bands. Separated LDH isoforms were visualized using an enzymatic-activity staining solution containing sodium lactate (3.24 mg/mL), nicotinamide adenine dinucleotide (NAD, 0.3 mg/mL), nitroblue tetrazolium (0.8 mg/mL) and phenazine methosulfate (0.167 mg/mL) (Sigma-Aldrich) dissolved in 0.01 M Tris (pH 8.5). LDH band densitometry was performed using a LI-COR Odyssey imager (Lincoln, NE).

### Quantitative measurement of LDH subunit expression

Purified AEC2s were lysed (150 mM NaCl; 50 mM Tris pH 8; 1% NP-40; 1 mM EDTA; 0.5% SDS; 1X protease inhibitor cocktail), then 3 µg of total protein per sample/gel were treated with 50 mM dithiothreitol before SDS-PAGE and western transfer to nitrocellulose as previously described [25]. Duplicate gels/blots were produced to separately probe with rabbit anti-LDHA or -LDHB antibodies (Proteintech, Rosemont, IL; cat. #s 19987 and 14824). Mouse anti-β Actin (Proteintech, cat #66009) monoclonal was used for both blots. Donkey anti-rab IRDye 800CW and donkey anti-mouse IRDye 680RD secondary antibodies (both LI-COR) were used to simultaneously visualize bands on a LI-COR Odyssey imager, with densitometry and normalization achieved using LI-COR Image Studio software. LDH subunit expression was normalized to β-actin for each sample. Furthermore, to ensure equivalent signals from both LDH antibodies, normalization was also obtained by running purified LDH3 tetramer (Aalto Scientific; composed of 2 LDHA and 2 LDHB subunits) on each gel as a standard.

### Experimental manipulation of LDHA expression

Targeted, siRNA-mediated knockdown of LDHA expression was performed. Briefly, purified human IPF AEC2s were seeded on rat-tail collagen coated 6-well plates in Opti-MEM reduced serum medium (Life Technologies) at 3 × 10^5^ cells/well. A validated, LDHA-specific Silencer siRNA (AM16708-113235; Ambion, Austin, TX) or a scrambled control siRNA (sc-37007; Santa Cruz Biotechnology, Dallas, TX) were separately transfected into cells using the lipofectamine RNAiMAX reagent (Life Technologies), according to the manufacturer’s instructions. After 48 h in culture, cells were detached, seeded onto XF96 plates and 24-h later, subjected to OCR and PPR measurements using a Seahorse Bioscience instrument. Cellular lysates were also harvested for both native gel electrophoresis and qPCR. RNA was extracted using an RLT Plus kit (Qiagen, Germantown, MD), and qPCR assays were performed as previously described [25], using LDHA (F, 5'-CCTGGGATCCAGTGTATAAATCC-3'; R, 5'-CCAAAGTAGTCACTGTTCAAGGT-3') or 18S rRNA (F, 5'-CCAGAGCGAAAGCATTTGCCAAGA-3'; R, 5'- TCGGCATCGTTTATGGTCGGAACT-3') primer pairs synthesized by Integrated DNA Technologies (Coralville, IA). Fold-changes of LDHA normalized to 18S rRNA were calculated using ΔΔCt analysis and values for triple replicates were averaged.

### Statistics

Calculations were performed using GraphPad Prism Software (San Diego, CA). Significant differences were assessed using Student’s t-Test or ANOVA. p values < 0.05 were considered significant. Error bars represent ± standard deviation. Additional statistical details for experiments are provided in figures or legends.

## Results

### Oxidative and glycolytic metabolism in AEC2 from human IPF lungs

**Fig. 1:**
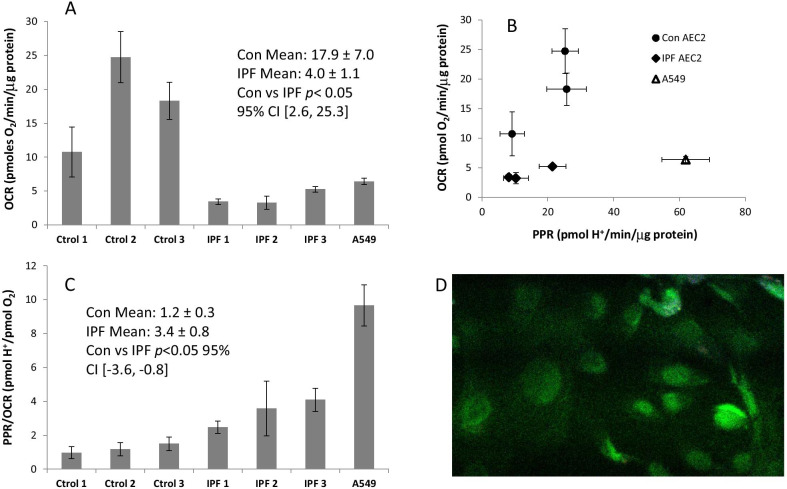
Oxidative and glycolytic metabolism in AEC2s from human IPF lungs. **A** Oxygen-consumption rates (OCR) compared between primary AEC2s from 3 non-fibrotic human lungs (con), AEC2s from 3 IPF transplant patients, and the A549 cell-line. Each AEC2 sample (or A549 cells) were analyzed in quadruplicate replicates. **B** OCR vs. proton-production rates (PPR) of the same AEC2 cells from *A*. **C** Comparisons of PPR/OCR ratios of the AEC2s. All values were normalized to total cellular protein. **D** Immunocytochemistry of IPF2 AEC2 showing staining for pro-SP-C (FITC or Green) to demonstrate primary cell purity

To test our hypothesis that mitochondrial metabolism is altered to a more glycolytic phenotype in primary human IPF AEC2s, we first examined oxidative and glycolytic function in primary cells isolated from IPF and control, non-fibrotic human donor lung tissue. Oxygen-consumption rates (OCR) and proton-production rates (PPR) were measured as indicators of oxidative and glycolytic metabolism, respectively. As a comparison, metabolic measurements of the highly-glycolytic, human lung adenocarcinoma cell-line A549 were also performed. As shown in Fig. 1, AEC2 from all individual IPF lung samples trended lower OCR (Fig. 1A) and similar PPR (Fig. 1B) compared to controls. Therefore, IPF AEC2 showed comparatively higher PPR/OCR ratios (Fig. 1C), indicating enhanced glycolytic vs. oxidative metabolism.

### LDH isoenzyme expression in AEC2 from human IPF lungs

**Fig. 2:**
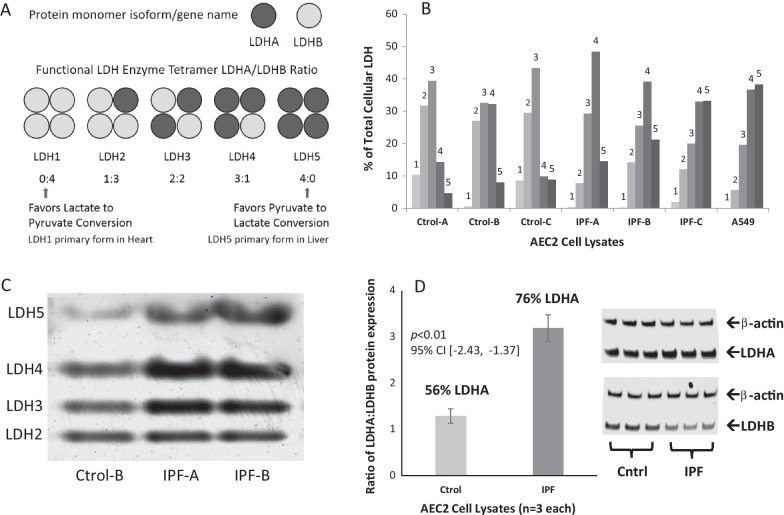
LDH isoenzyme expression in AEC2 from fibrotic IPF vs. non-fibrotic control human lungs. **A** Schematic of LDHA and LDHB subunit compositions in the 5 isoforms of the active LDH tetramer. **B** Non-denatured lysates from purified AEC2s of 3 control/non-fibrotic and 3 IPF human lung samples were separated by native gel electrophoresis and subjected to colorimetric staining for LDH activity to measure the % of each isoenzyme tetramer (LDH1 to 5) comprising total LDH in each sample. The A549 cell-line was also analyzed for comparison. **C** Colorimetric LDH staining after native PAGE showing separated LDH tetramer isoforms of three AEC2 sample lysates from *B*. **D** Lysates from AEC2s purified from 3 control/non-fibrotic and 3 IPF lungs were analyzed by SDS-PAGE and western blot using anti-LDHA and –LDHB antibodies. Immunoblots and average LDHA:LDHB ratio of 3 samples from each lung type are shown. LDH subunit expression was normalized to β-actin expression

Based on the differences in metabolic activity observed between primary AEC2s from non-fibrotic and IPF lungs, and in conjunction with our previous report that AEC2s preferentially utilize lactate as a metabolic substrate [18], we next examined whether this metabolic shift in IPF AEC2s is associated with changes in LDH isoenzyme tetramer composition associated with forms favoring pyruvate to lactate conversion (Fig. 2; i.e., higher LDHA to LDHB subunit ratios and, therefore, more LDH4 and 5 forms of the active tetramers; illustrated in Fig. 2A). Native gel electrophoresis was used to separate the 5 intact tetramer isoforms based on their electrical charge. By this approach, we observed differences in the relative representation of LDH tetramer isoforms in IPF AEC2 compared to those in controls, with LDH4 and LDH5 tetramers combined representing > 60% of the total active LDH pool in the IPF AEC2s (Fig. 2B). Overall, this distribution of LDH isoforms in IPF AEC2 was more similar to the that of the highly-glycolytic A549 cell line. Conversely, AEC2 from non-fibrotic lungs expressed relatively more LDH2 and 3 isoforms (Figs. 2B & C). Specifically, mean percentages of LDH isoforms in control AEC2 vs. IPF AEC2, respectively, were: LDH1 6.4% vs. 0.8%, p = 0.13; LDH2 29.4% vs 11.3%, p = 0.002; LDH3 38.4% vs. 24.9%, p = 0.03; LDH4 18.7% vs. 40.2%, p = 0.05; LDH5 7.1% vs. 23.0%, p = 0.04.

To directly corroborate these results with cellular protein expression of the LDH subunits, 3 representative samples each of purified AEC2 (control and IPF lungs) were subjected to SDS-PAGE and analyzed for both LDHA and LDHB subunit expression using quantitative protein immunoblotting (Fig. 2D). The results showed that the ratio of LDHA:LDHB subunit expression was significantly higher in AEC2s from IPF lungs vs. controls. Immunocytochemistry of LDH subunits within the cells was performed but was found to be less sensitive quantitatively compared to analyses of lysates by Western blotting or native LDH colorimetric staining (data not shown).

### Relationship of LDH isoenzyme expression on oxidative and glycolytic metabolism in AEC2s from human IPF lungs

**Fig. 3:**
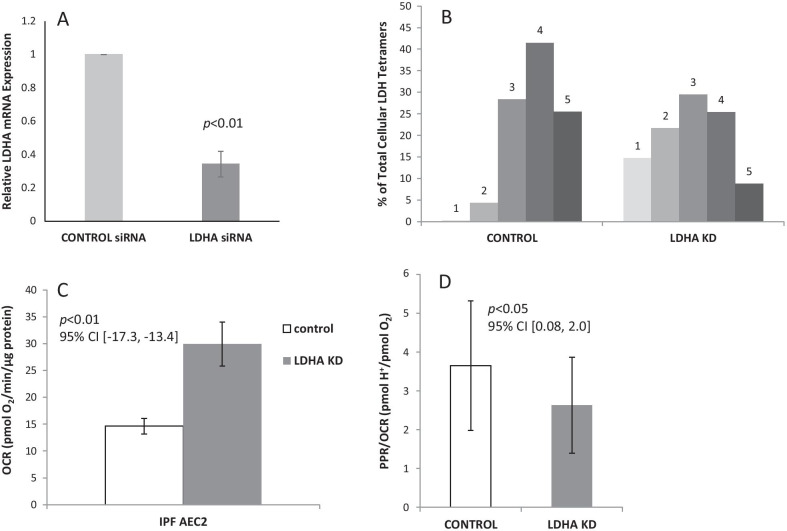
Relationship of LDH isoenzyme expression to oxidative and glycolytic metabolism in human IPF AEC2s. Purified AEC2s from tissues sections of three IPF patients were subjected to targeted siRNA transfection to knockdown LDHA expression (LDHA KD; compared to cells transfected with a scrambled siRNA control). **A** LDHA mRNA expression in IPF AEC2 cells 72 h after transfection was measured using qPCR. Fold-changes of LDHA normalized to 18S rRNA were calculated using ΔΔCt analysis and values for triple replicates were averaged; relative LDHA expression in scrambled siRNA control cells was set as 1.0. **B** Non-denatured lysates from IPF AEC2 72 h after transfection were separated by native gel electrophoresis and subjected to colorimetric staining for LDH activity to measure the % of each isoenzyme tetramer (LDH1 to 5) comprising total LDH. **C** OCR rates were measured and compared between these transfected IPF AEC2s cultured in the presence of both glucose and lactate. **D** The ratio of PPR to OCR in these transfected cells. All values were normalized to total cellular protein

Based on the comparatively higher PPR/OCR ratios and associated higher LDHA:LDHB subunit expression ratios in AEC2s from IPF lungs compared to non-fibrotic controls, we next investigated whether altered LDH isoenzyme tetramer composition could be a driver of the glycolytic shift in IPF AEC2s. For these experiments, primary AEC2s purified from a human IPF lung tissues (IPF-B from Fig. 2), which expressed a high LDHA:LDHB ratio, were subjected to targeted siRNA-mediated knockdown of LDHA subunit expression before measuring effects on cellular metabolism and LDH tetrameric forms. Compared to IPF AEC2s transfected with a scrambled siRNA control, those transfected with LDHA knockdown (KD) siRNA demonstrated a 66% reduction in LDHA mRNA expression as measured by qPCR (Fig. 3A). Consequently, this LDHA knockdown dramatically changed the relative proportions of tetrameric LDH protein isoforms in the cells, with increases in LDH2 and 3 forms (containing more LDHB subunits) and reductions in the LDHA-prevalent LDH4 and 5 forms (Fig. 3B). As shown in Fig. 3C, this LDHA KD not only resulted in IPF AEC2 with an LDH tetramer expression pattern more closely resembling that of representative AEC2s from non-fibrotic lungs (compare Fig. 2B), but also significantly increased IPF AEC2 OCR to levels similar to those non-fibrotic AEC2 (compare Fig. 1A). Overall, this LDH tetramer shift after LDHA knockdown was correlated with a reduction in PPR/OCR ratio in the IPF AEC2s compared to scrambled siRNA controls (Fig. 3D). Thus, manipulation of LDH isomer expression per se in IPF AEC2s drove a metabolic shift to AEC2s resembling those from non-fibrotic lungs.

## Discussion

We have previously shown that healthy AEC2s are highly oxidative, preferentially utilize lactate as a metabolic substrate for mitochondrial ATP production, and that pharmacological inhibition of LDH significantly alters metabolism in these cells [18]. In this current study, we demonstrated that AEC2s from representative IPF lung patient specimens had a distinct bioenergetic profile compared to those obtained from non-fibrotic patient lungs. IPF AEC2s had comparatively lower OCR than those from non-fibrotic lungs, with comparatively higher PPR/OCR ratios, indicating enhanced glycolytic vs. oxidative metabolism. Collectively, our results strongly suggest that these bioenergetic profiles are directly due to differences in LDH isoenzyme distribution between non-fibrotic and IPF lung AEC2s. LDH isoenzyme distribution in IPF AEC2s was characterized by high percentages of forms favoring pyruvate to lactate conversion (i.e., more LDH4 and 5 forms of the active tetramers), while non-fibrotic lung AEC2s were characterized by more LDH2 and 3 forms. These differences in LDH tetramer were due to an increase in LDHA subunit expression in IPF lung AEC2s. Adding credence to this model, experimental knockdown of the LDHA protein subunit expression in IPF AEC2s resulted in cells that more closely resembled the bioenergetic profile and LDH isoenzyme expression of AEC2s from non-fibrotic lungs. Overall, both the bioenergetic profile and LDH isoenzyme distribution of IPF AEC2s resembled that of the highly glycolytic, AEC2-like adenocarcinoma cell line A549 more so than that of non-fibrotic AEC2s.

The alveolar epithelium is predominantly comprised of two cell types: alveolar epithelial type I (AEC1) and AEC2s. AEC1s are essential for gas exchange, while the smaller, cuboidal AEC2 play central roles in lung homeostasis and pathophysiology from premature infants to adults [29]. AEC2s primarily function in production and secretion of pulmonary surfactant, processes demanding significant amounts of energy [30]. Additionally, AEC2 have important roles in innate immunity and are multipotent stem cells that can terminally-differentiate into AEC1 cells [31–33]. The physiology of AEC2s has particular clinical relevance, as pulmonary surfactant dysfunction is a hallmark of numerous lung diseases, including IPF and respiratory distress syndrome [34–37]. These important roles underscore how injury to either or AEC1 and/or AEC2 injury could be an underlying cause of, and prevention of repair of the epithelium in IPF, while feedback loop interactions between dysfunctional AEC2s and activated fibroblasts, including promoting epithelial-to-mesenchymal transition wherein the AEC2 also acquire a fibroblast phenotype, likely accelerate disease progression [9, 11–13, 38, 39].

Recent reports of processes contributing to myofibroblast activation in IPF have highlighted the importance of lactate balance in the lung and support our premise that dysregulated AEC2 cellular metabolism is a major driver of IPF progression. Increased lactate generation was associated with enhanced tissue expression of LDH-5, the isoform most strongly favoring pyruvate-to-lactate conversion. Altered expression was localized roughly to the epithelium near fibrotic centers, though the specific cell(s) responsible were not identified [14].

Our results in this study are consistent with the hypothesis that IPF AEC2s exhibit dysfunctional lactate utilization. Many factors other than concentration of LDH protein per se determine whether lactate is generated or consumed, one being the LDH isoenzyme that is expressed. LDH is a tetrameric protein composed of LDHA (LDHm) and LDHB (LDHh) subunits, and five different LDH isoenzymes exist (see Fig. 2A). LDH1 through LDH5 represent a spectrum of isoenzymes, with LDH1 (composed entirely of LDHB; e.g., found in the heart) favoring lactate oxidation and LDH5 (composed entirely of LDHA; found in the liver and skeletal muscle) strongly favoring pyruvate reduction to generate lactate [19, 20]. Thus, the characteristic distribution of LDH4 and 5 forms we found in AEC2s from fibrotic lungs would likely be associated with highly glycolytic production and export of lactate. Conversely, the distribution of LDH2 and 3 tetramers characteristic of AEC2s from non-fibrotic lungs would favor interconversion of pyruvate and lactate depending on substrate availability and would be associated with cells that use lactate, if available, as a metabolic substrate for oxidative phosphorylation. These results are consistent with our premise that IPF AEC2s exhibit dysfunctional lactate utilization, which would reduce OxPhos, limiting bioenergetics and intermediary pathways to maintain surfactant production (i.e., loss of functional AEC2 phenotype), and increase lactate production in the alveolar microenvironment and/or buildup of lactate in adjacent cells. These observations are also consistent with previous reports that describe lactate accumulation in IPF tissue and alterations in metabolic pathways when compared to normal lung samples [14, 40].

Increased expression of LDHA in IPF AEC2 could be directly related to many other factors known to be dysregulated in IPF, including TGFβ and mTOR activation and downregulation of numerous regulatory microRNAs, including those of the miR200 family [13, 15–17]. These factors are also closely involved in promoting the glycolytic production of lactate (Warburg effect) seen in many cancers [41–44]. Increased lactate production in IPF through changes in LDHA expression is clearly similar to that seen in tumorigenesis.

To provide a more representative model for identifying therapeutic targets and to study the role of alveolar epithelium in the pathogenesis of IPF, we utilized primary AEC2s isolated form explant lung tissues of transplant patients. By comparing IPF AECs to non-fibrotic patient AECs, we discovered distinct bioenergetic profiles and LDH isoenzyme expression patterns. Extending these basic findings to clinical translation, we suggest that glycolytic metabolic enzymes, in particular LDH, represent a therapeutic target to combat the pro-fibrotic environment that drives myofibroblast differentiation and synthetic function in IPF. The concept of therapeutic targeting of glycolysis to slow or reverse disease processes is not necessarily novel; it has been well explored as a strategy in cancer chemotherapy. In fact, specific targeting of the LDH5 isoform has been a focus of intense interest in cancer therapeutics research for many years [45–48]. Here, we utilized RNAi to target LDH isoform distribution by reducing LDHA subunit expression in IPF AEC2s. This manipulation of LDHA effectively reversed the OxPhos and OCR/PPR bioenergetic profile characteristic of AECs from IPF lungs and restored the profile characteristic of those from non-fibrotic lungs. There are currently multiple small-molecule inhibitors of LDH5, under investigation for efficacy in cancer and Parkinson’s disease [49–51], which we suggest could be similarly effective at re-establishing normal AEC2 metabolism in IPF.

In summary, this work provides evidence that IPF is, at least in part, a disease driven by metabolic dysregulation. We show fundamental changes in AEC2 metabolism with potential to cause lactic acid imbalance in lung tissue and contribute to a pro-fibrotic extracellular environment. This furthermore suggests a new avenue for therapeutic intervention. Targeting metabolic pathways to reverse the pathological shifts leading to lactic acid buildup may prevent some of the downstream changes driving progressive fibrosis. Continued investigation into these pathogenic mechanisms and how they may be targeted in human patients, even by existing pharmaceutical agents, will be of critical importance for the future of IPF treatment. Based on our findings, our major premise is that dysregulated AEC2 cellular metabolism is a major driving cause of IPF and that manipulation of lactate metabolism in particular may be a means to intervene and possibly reverse this fatal disease.

## Conclusions

The results of this study are consistent with the concept that altered lactate metabolism may be an underlying feature of AEC2 dysfunction in IPF and may be a novel and important target for therapeutic treatment. As exemplified in the study presented herein, viable primary cells isolated from pathological lung tissues will be essential to progressing toward patient stratification and precision medical therapeutics.

## Data Availability

All data generated or analysed during this study are included in this published article.

## Abbreviations

AEC2: Alveolar type II epithelial cells
IPF: Idiopathic pulmonary fibrosis
KD: SiRNA-mediated knockdown
LDH: Lactate dehydrogenase
LDHA/B: LDH A or B subunits
MUSC: Medical University of South Carolina
OCR: Oxygen-consumption rate
PPR: Proton-production rate
